# A comparative analysis of the performance of leading large language models on the endodontics section of the dentistry specialization exam in Türkiye

**DOI:** 10.1371/journal.pone.0350457

**Published:** 2026-06-01

**Authors:** Beyhan Başkan, Hatice Kübra Başkan, Nevzat Koç

**Affiliations:** 1 Department of Endodontics, Faculty of Dentistry, Kahramanmaraş Sütçü İmam University, Kahramanmaras, Türkiye; 2 Department of Pediatric Dentistry, Faculty of Dentistry, Kahramanmaraş Sütçü İmam University, Kahramanmaras, Türkiye; 3 Department of Endodontics, Faculty of Dentistry, Niğde Ömer Halisdemir University, Nigde, Türkiye; Federal University of Santa Maria: Universidade Federal de Santa Maria, BRAZIL

## Abstract

**Objective:**

This study aimed to evaluate and compare the performance of eight contemporary LLMs on the endodontics section of the DUS, assessing their accuracy in both theoretical knowledge and simulated clinical scenarios from historical exam data.

**Methods:**

The performance of eight different large language models (Claude 4, DeepSeek V3, Gemini 2.5 Pro, ChatGPT-4o, ChatGPT-5, Grok 4, LLaMA 4, and Perplexity) was evaluated using 127 multiple-choice endodontics questions from the Specialization Exam in Dentistry (DUS) administered by the Student Selection and Placement Center (ÖSYM) between 2012 and 2021. The models’ responses were compared against the official answer keys. Statistical analyses were performed using Pearson’s chi-square and McNemar tests, with a significance level of α = 0.05.

**Results:**

Significant differences existed among LLMs in overall accuracy (p < 0.001). Gemini 2.5 Pro achieved the highest accuracy (90.6%), outperforming ChatGPT-4o (61.4%) and LLaMA 4 (71.7%). In Clinical Practice Questions (CPQ), Gemini 2.5 Pro (93.9%) surpassed ChatGPT-4o (57.6%; p = 0.019). For General Knowledge and Concept Questions (GKCQ), Gemini 2.5 Pro (89.4%), Grok 4 (85.1%), and DeepSeek V3 (84.0%) exceeded ChatGPT-4o (62.8%; p < 0.001). No significant intra-model differences emerged between CPQ and GKCQ performance (p > 0.05).

**Conclusion:**

Contemporary LLMs demonstrate substantial competence in endodontic knowledge, with Gemini 2.5 Pro excelling in both theoretical and clinical queries. However, significant performance variability across models (61.4%−90.6%) and the complexity of retrieving and resolving clinical exam queries necessitate domain-specific optimization and expert oversight for reliable integration into dental education and practice.

## Introduction

The rapid evolution of artificial intelligence (AI) has initiated a transformative era in healthcare, fundamentally changing the landscape of medical and dental practice [1,2]. At the forefront of this revolution are Large Language Models (LLMs), sophisticated AI systems designed to comprehend and generate human-like text by processing vast datasets through deep learning architectures [3]. These models have demonstrated remarkable potential in various high-stakes domains, including passing medical licensing examinations and assisting in complex clinical decision-making [4,5]. In the field of dentistry, the integration of AI is already proving beneficial in diagnostic tasks, treatment planning, and dental education [6].

The competence of LLMs in dental knowledge is assessed through various tools such as specialized question banks, board-style practice exams, and clinical cases. In this process, national licensing examinations are considered a critical benchmark for professional competency. Studies have shown that models can perform at a level competitive with on the United States Medical Licensing Examination (USMLE) and various national dental boards [4,7,8]. Current evaluations reveal that models like Claude, Gemini, and ChatGPT-4 have surpassed their predecessors in basic sciences, but they still exhibit limitations in complex questions requiring clinical reasoning. The rapid pace of artificial intelligence development necessitates continuous and comparative assessments to ensure clinical safety. The reliability of AI-generated content in a clinical context requires expert supervision and a rigorous verification process [5,9].

In the Türkiye dental education system, the Dentistry Specialization Exam (DUS) is the definitive assessment for clinical expertise. Evaluating how current LLMs perform on DUS Endodontics questions is critical not only for assessing their utility as educational aids, bearing in mind the distinction between data recall and clinical decision-making. However, the integration of these models into specialized fields like endodontics is complicated by their inherent technical limitations. Despite their ability to accelerate information retrieval, LLMs are still prone to “hallucinations”—generating plausible but incorrect clinical advice—and may reflect biases within their training datasets [2,10]. Recent literature emphasizes that such inaccuracies in endodontic contexts can lead to incorrect diagnoses or flawed treatment planning, necessitating a rigorous verification of AI-generated outputs against established clinical standards [10,11]. These persistent challenges underscore the need for a nuanced and comparative analysis of AI’s performance before it can be safely integrated into high-stakes endodontic practice.

This research evaluates the knowledge level and text-based clinical problem-solving capabilities of today’s leading LLMs (Claude 4, DeepSeek V3, Gemini 2.5 Pro, ChatGPT-4o, ChatGPT-5, Grok 4, LLaMA 4, and Perplexity) specifically in the field of DUS Endodontics. By comparing model performance on General Knowledge and Concept Questions (GKCQ) and Clinical Practice Questions (CPQ), we aim to identify the potential and limitations of these technologies in dental education and clinical practice. Specifically, we hypothesized that: (i) there would be statistically significant differences in overall accuracy rates among the LLMs, (ii) more recent models would demonstrate superior performance, and (iii) LLMs would perform differently on clinical practice questions versus general knowledge questions.

## Materials and methods

This study did not require ethics committee approval as it was not conducted on human or animal subjects. All data used in the research consisted of outputs from artificial intelligence models accessed via public platforms. No patient data or personally identifiable information was used during the study.

Sample size was determined through power analysis using G*Power software (v3.1.9.7), assuming a significance level of α = 0.05, statistical power of 1-β = 0.80, and medium effect size (d = 0.5), yielding a minimum requirement of 97 questions. As DUS questions from 2021 onwards have not been publicly released, 130 multiple-choice questions from the endodontics section of DUS examinations conducted by the Student Selection and Placement Center (ÖSYM) between 2012 and 2021 were included in this study.

LLMs evaluated in our study were: Claude 4 (Anthropic, San Francisco, USA), DeepSeek V3 (DeepSeek, Hangzhou, Zhejiang, China), Gemini 2.5 Pro (Google, Mountain View, California, USA/ DeepMind, Londra, England), ChatGPT-4o (OpenAI, San Francisco, California, USA), ChatGPT-5 (OpenAI, San Francisco, California, USA), Grok 4 (xAI, Palo Alto/ Bay Area, USA), LLaMA 4 (Meta Platforms, Menlo Park/ Bay Area, California, USA), Perplexity (Perplexity AI, San Francisco, California, USA) The development companies and release dates for each of these models are detailed in Table 1. All data collection and LLM queries were performed in a single session for each LLM on October 15, 2025.

**Table 1:** Production dates and affiliated companies of the Large Language Models (LLMs) used in our study.

Artificial Intelligence Model	Company	Release Date
Claude 4 (Opus 4 & Sonnet 4)	Anthropic	May 2025
DeepSeek V3	DeepSeek	March 2025
Gemini 2.5 Pro	Google	Early 2025 (Preview)/ June 2025 (Major Update)
ChatGPT-4o	OpenAI	May 2024
ChatGPT-5	OpenAI	August 2025
Grok 4 (xAI)	xAI	July 2025
LLaMA 4	Meta Platforms	April 2025
Perplexity	Perplexity AI	August 2022

**Fig 1:**
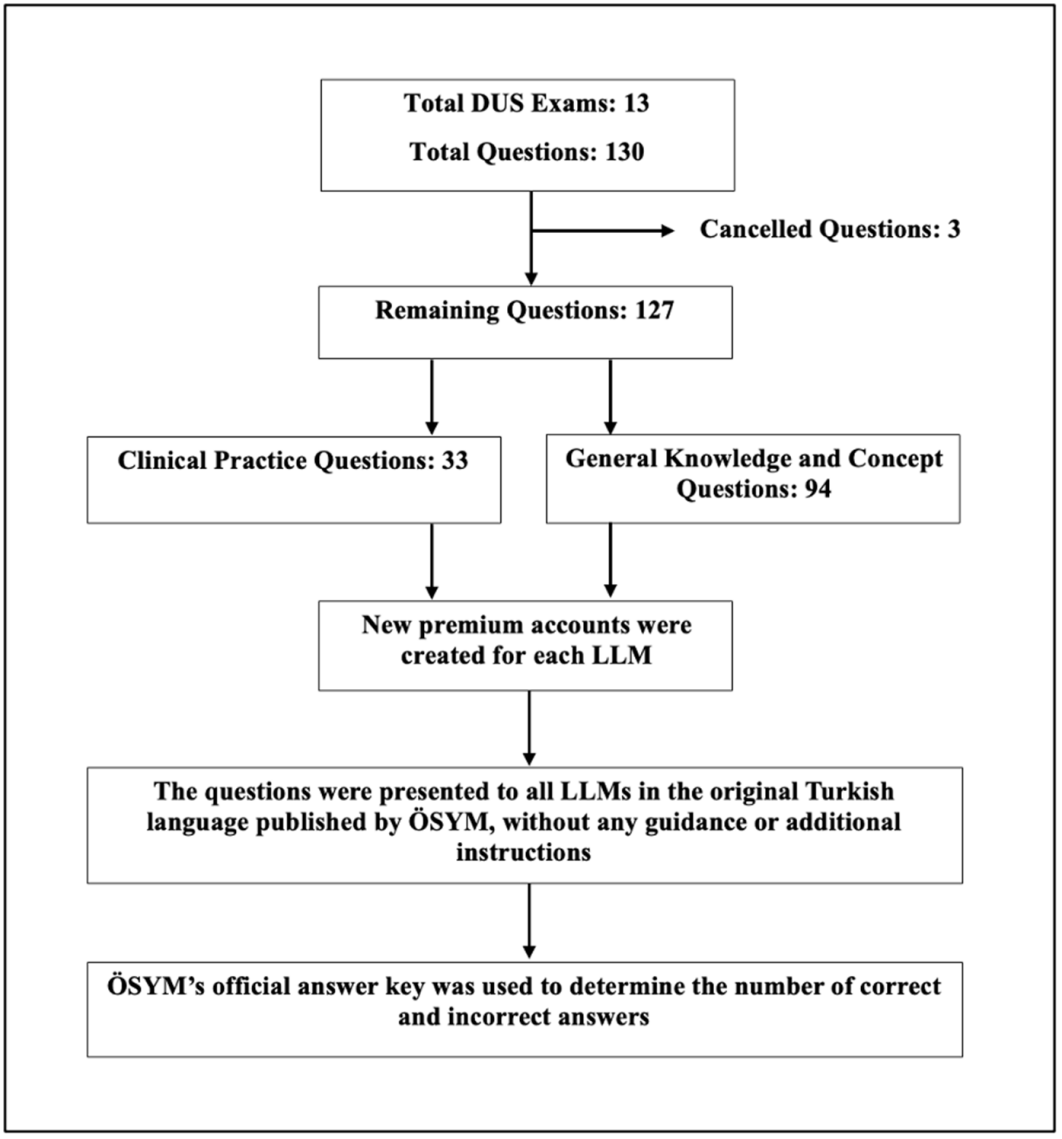
A flowchart illustrates the division of a total of 127 questions from the DUS Endodontics exam into two main categories: General Knowledge and Concept Questions (GKCQ, n = 94) and Clinical Practice Questions (CPQ, n = 33), along with the implementation steps. LLMs: Large Language Models; DUS: Dentistry Specialization Exam; ÖSYM: Student Selection and Placement Center; GKCQ: General Knowled‌‌ge and Concept Questions; CPQ: Clinical Practice Questions.

In this study, 130 multiple-choice endodontics questions, each with five answer options, were used from the DUS examinations (2012–2021) published by ÖSYM [12]. Questions from 2022 to 2025 were not included as they have not been publicly released. Three cancelled questions from the exams included were also excluded, resulting in a total of 127 questions for analysis. To better evaluate the models’ performance on different question types, the questions were independently categorized by two endodontists with 6 and 8 years of clinical experience. Disagreements were resolved through consensus discussion into: GKCQ (n = 94) and CPQ (n = 33) (Fig 1).

For each LLM, new accounts were created and a premium subscription was purchased. The questions were presented to all LLMs in the original Türkiye language published by ÖSYM, without any additional instructions, system-specific commands, or guidance. Each question was directly copied and pasted into the LLMs’ chat interfaces in its original format, including all five answer options, without any additional instructions or prompt engineering. To prevent learning biases and iterative performance improvements, the responses were requested and recorded only once. This single-query, zero-shot approach was chosen to establish a baseline performance measure reflecting a typical user interaction without iterative refinement. The answers were then classified as correct or incorrect by three specialists (two endodontists and one pediatric dentistry specialist) using ÖSYM’s official answer keys. For each LLM, the distribution of correct and incorrect responses was analyzed separately for CPQ, GKCQ, and total questions.

### Statistical analysis

Statistical Package for Social Sciences (SPSS) Windows version 27 (SPSS Inc. Chicago, IL, USA) software program was used for data analysis in the study. Descriptive statistics (frequencies, percentages) of the data were presented. To compare performance between different LLMs (which represent independent groups), Pearson’s Chi-square test was used to assess differences in overall accuracy, as well as in CPQ and GKCQ subsets separately. For performance comparisons within each individual LLM (i.e., paired comparison of CPQ vs. GKCQ accuracy for the same model), the McNemar test was applied, as it is designed for paired nominal data (correct/incorrect responses from the same subject on two related question types). Response pattern homogeneity across models was additionally assessed through hierarchical clustering analysis using a distance metric with complete linkage. In pairwise post-hoc comparisons following significant Chi-square results, Bonferroni correction was applied to control Type I error, and corrected p-values were reported. The statistical significance level was set at p < 0.05 for all analyses.

## Results

**Fig 2:**
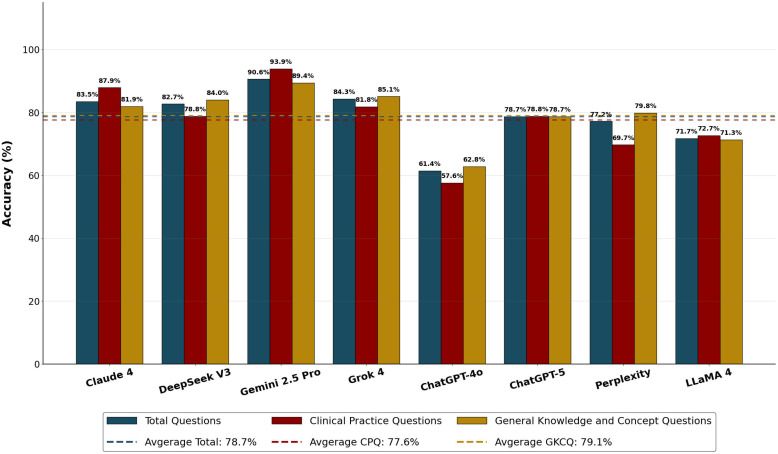
This graph illustrates the accuracy percentages of Large Language Models (LLMs) across three categories: total questions (blue bars), clinical practice questions (CPQ, red bars), and general knowledge and conceptual questions (GKCQ, gold bars). The dashed horizontal lines represent the overall average performance of all evaluated models within each respective category. LLMs: Large Language Models; CPQ: Clinical Practice Questions; GKCQ: General Knowledge and Concept Questions.

A statistically significant difference was observed among LLMs in correctly answering all questions (χ² (7) = 42.521, p < 0.001). Gemini 2.5 Pro (90.6%; 115/127) had a statistically significantly higher accuracy rate than ChatGPT-4o (61.4%; 78/127) and LLaMA 4 (71.7%; 91/127). Claude 4 (83.5%; 106/127), DeepSeek V3 (82.7%; 105/127), and Grok 4 (84.3%; 107/127) had statistically significantly higher accuracy rates than ChatGPT-4o (61.4%) (Table 2). The accuracy percentages of each model across total, CPQ, and GKCQ categories are summarized in Fig 2.

**Table 2:** Comparison of the overall accuracy rates of LLMs on the Dentistry Specialization Exam (DUS) Endodontics questions (n = 127).

LLMs		Total Questions		χ² (7)	p
n	%
Claude 4	Correct	106^ab^	83.5	42.521	<0.001*
	Incorrect	21	16.5		
DeepSeek V3	Correct	105^ab^	82.7		
	Incorrect	22	17.3		
Gemini 2.5 Pro	Correct	115^a^	90.6		
	Incorrect	12	9.4		
Grok 4	Correct	107^ab^	84.3		
	Incorrect	20	15.7		
ChatGPT-4o	Correct	78^c^	61.4		
	Incorrect	49	38.6		
ChatGPT-5	Correct	100^abc^	78.7		
	Incorrect	27	21.3		
Perplexity	Correct	98^abc^	77.2		
	Incorrect	29	22.8		
LLaMA 4	Correct	91^bc^	71.7		
	Incorrect	36	28.3		
Average	Correct	100	78.7		
	Incorrect	27	21.3		

Different superscript letters (a, b, c) within the same column indicate a statistically significant difference between groups according to Bonferroni-adjusted post-hoc analysis (p < 0.05).

LLMs: Large Language Models; χ²: Pearson’s Chi-square test value

* p < 0.05

A statistically significant difference was observed among LLMs in correctly answering Clinical Practice Questions (χ² (7) = 16.741, p = 0.019). Gemini 2.5 Pro (93.9%; 31/33) had a statistically significantly higher accuracy rate than ChatGPT-4o (57.6%; 19/33). The performances of other LLMs were not statistically significantly different from each other (Table 3).

**Table 3:** Comparison of LLMs’ performance on Clinical Practice Questions (CPQ, n = 33).

LLMs		Clinical Practice Questions		χ² (7)	p
n	%
Claude 4	Correct	29^ab^	87.9	16.741	0.019*
	Incorrect	4	12.1		
DeepSeek V3	Correct	26^ab^	78.8		
	Incorrect	7	21.2		
Gemini 2.5 Pro	Correct	31^a^	93.9		
	Incorrect	2	6.1		
Grok 4	Correct	27^ab^	81.8		
	Incorrect	6	18.2		
ChatGPT-4o	Correct	19^b^	57.6		
	Incorrect	14	42.4		
ChatGPT-5	Correct	26^ab^	78.8		
	Incorrect	7	21.2		
Perplexity	Correct	23^ab^	69.7		
	Incorrect	10	30.3		
LLaMA 4	Correct	24^ab^	72.7		
	Incorrect	9	27.3		
Average	Correct	25.6	77.6		
	Incorrect	7.4	22.4		

Different superscript letters (a, b) within the same column indicate a statistically significant difference between groups according to Bonferroni-adjusted post-hoc analysis (p < 0.05).

LLMs: Large Language Models; χ²: Pearson’s Chi-square test value

* p < 0.05

A statistically significant difference was observed among LLMs in correctly answering GKCQ (χ² (7) = 28.585, p < 0.001). Gemini 2.5 Pro (89.4%; 84/94), Grok 4 (85.1%; 80/94), and DeepSeek V3 (84.0%; 79/94) had statistically significantly higher accuracy rates than ChatGPT-4o (62.8%; 59/94) and LLaMA 4 (71.3%; 67/94). Claude-4 (81.9%; 77/94), Perplexity (79.8%; 75/94), and ChatGPT-5 (78.7%; 74/94) had statistically significantly higher accuracy rates than ChatGPT-4o (62.8%; 59/94) (Table 4).

**Table 4:** Comparison of LLMs’ performance on General Knowledge and Concept Questions (GKCQ, n = 94).

LLMs		General Knowledge and Concept Questions		χ² (7)	p
n	%
Claude 4	Correct	77^ab^	81.9	28.585	<0.001*
	Incorrect	17	18.1		
DeepSeek V3	Correct	79^a^	84		
	Incorrect	15	16		
Gemini 2.5 Pro	Correct	84^a^	89.4		
	Incorrect	10	10.6		
Grok 4	Correct	80^a^	85.1		
	Incorrect	14	14.8		
ChatGPT-4o	Correct	59^c^	62.8		
	Incorrect	35	37.1		
ChatGPT-5	Correct	74^ab^	78.7		
	Incorrect	20	21.3		
Perplexity	Correct	75^ab^	79.8		
	Incorrect	19	20.2		
LLaMA 4	Correct	67^bc^	71.3		
	Incorrect	27	28.7		
Average	Correct	74.3	79.1		
	Incorrect	19.7	20.9		

Different superscript letters (a, b, c) within the same column indicate a statistically significant difference between groups according to Bonferroni-adjusted post-hoc analysis (p < 0.05).

LLMs: Large Language Models; χ²: Pearson’s Chi-square test value

* p < 0.05

No statistically significant difference was found between the correct response rates of each LLM for CPQ and GKCQ (Claude 4: 87.9% vs 81.9%, p = 0.427; DeepSeek V3: 78.8% vs 84.0%, p = 0.492; Gemini 2.5 Pro: 93.9% vs 89.4%, p = 0.439; Grok 4: 81.8% vs 85.1%, p = 0.655; ChatGPT-4o: 57.6% vs 62.8%, p = 0.598; ChatGPT-5: 78.8% vs 78.7%, p = 0.993; Perplexity: 69.7% vs 79.8%, p = 0.234; LLaMA 4: 72.7% vs 71.3%, p = 0.873) (Table 5).

**Table 5:** Intra-model comparison of accuracy rates between Clinical Practice and General Knowledge and Concept Questions for each LLM.

LLMs	Question Types	Correct/Incorrect (n)	%	p
Claude 4	CPQ	29/33	87.9	0.427
	GKCQ	77/94	81.9	
DeepSeek V3	CPQ	26/33	78.8	0.492
	GKCQ	79/94	84	
Gemini 2.5 Pro	CPQ	31/33	93.9	0.439
	GKCQ	84/94	89.4	
Grok 4	CPQ	27/33	81.8	0.655
	GKCQ	80/94	85.1	
ChatGPT-4o	CPQ	19/33	57.6	0.598
	GKCQ	59/94	62.8	
ChatGPT-5	CPQ	26/33	78.8	0.993
	GKCQ	74/94	78.7	
Perplexity	CPQ	23/33	69.7	0.234
	GKCQ	75/94	79.8	
LLaMA 4	CPQ	24/33	72.7	0.873
	GKCQ	67/94	71.3	

LLMs: Large Language Models; CPQ: Clinical Practice Questions; General Knowledge and Concept Questions: GKCQ; McNemar test was applied for within-model comparisons between Clinical Practice and General Knowledge questions. No statistically significant differences were found for any LLM (p > 0.05).

**Fig 3:**
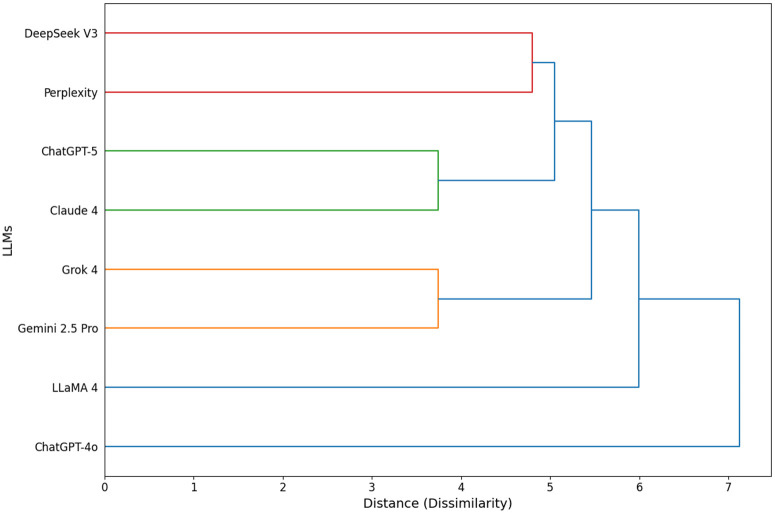
Hierarchical Clustering Dendrogram – The vertical axis represents the language models being evaluated, while the horizontal axis represents the rate of distinctiveness (dissimilarity) based on the Euclidean distance between the models. Colored lines (red, green, orange, blue) indicate s‌‌ubgroup clusters. The position of each merge point (node) on the horizontal axis reflects the degree of stylistic and structural similarity between models; as the distance decreases, the similarity between response patterns increases.

Hierarchical Clustering Dendrogram reveals significant insights into the similarities among LLM response patterns (Fig 3). Grok 4 and Gemini 2.5 Pro emerge as the most closely aligned pair, followed by GPT-5/Claude 4 and DeepSeek V3/Perplexity. These models form a consistent supercluster through sequential merging: GPT-5/Claude 4 first merges with DeepSeek V3/Perplexity, then Grok 4/Gemini 2.5 Pro joins—indicating shared response logic across all six models. While LLaMA 4 diverges distinctly, GPT-4o stands out as the most pronounced outlier (distance >7) and demonstrates fundamentally unique response patterns—even compared to its successor, GPT-5. This highlights GPT-4o’s exceptional behavioral divergence within the LLM landscape.

## Discussion

The annual DUS in Türkiye presents significant challenges through its integration of theoretical knowledge and clinical problem-solving. Top candidates typically achieve 105−115 correct answers out of 120, highlighting the exam’s rigor. Our study demonstrates that contemporary LLMs exhibit substantial competence in this demanding context, with Gemini 2.5 Pro emerging as the highest performer, significantly outperforming ChatGPT-4o and LLaMA 4. This performance hierarchy—Gemini 2.5 Pro > Grok 4/Claude 4/DeepSeek V3 > ChatGPT-5/Perplexity > LLaMA 4 > ChatGPT-4o—validates our hypothesis that more recent models generally demonstrate superior capabilities. The superior performance of Gemini 2.5 Pro observed in our study is consistent with recent findings in other dental specialties. In a comparative evaluation focusing on oral and maxillofacial disorders, Gemini was found to achieve the highest diagnostic scores among evaluated models, showcasing its potential as a complementary tool for clinical reasoning [13]. This alignment across different studies reinforces the potential of advanced LLMs to handle complex, case-based scenarios in dentistry, although their performance still remains sensitive to case difficulty.

Our finding of no significant intra-model differences between CPQ and GKCQ suggests that LLMs can effectively translate theoretical knowledge to text-based clinical scenarios within structured multiple-choice contexts, as exemplified by Gemini 2.5 Pro’s exceptional CPQ performance. However, this capability exhibits critical nuances influenced by model architecture, question design, and specialty-specific factors. First, model dependency is evident as Gemini 2.5 Pro dominates clinical queries while Perplexity lags at 69.7%. Second, question-type sensitivity emerges in Çekiç and Tavşan’s [14] endodontics study, where LLM accuracy dropped significantly (p < 0.05) on combination-type MCQs versus standard MCQs. Similarly, Tosun and Yılmaz [15] reported ChatGPT-4’s prosthodontics accuracy plummeted from 78.6% for knowledge-based questions to 45.5% for case-based ones. Third, specialty-specific barriers persist, with Sismanoglu and Capan [16] identifying endodontics and orthodontics as LLMs’ weakest areas, while Özbay et al. [17] noted high misinformation rates (27.5%) in open-ended clinical queries. The observed CPQ/GKCQ parity likely reflects the text-simulated nature of DUS clinical scenarios, which lack real-world complexities such as radiographic interpretation and may benefit from domain-specific architectural optimization. Contradicting our parity finding, Yılmaz et al. [18] observed significant performance variations (p = 0.034) in oral pathology case-based questions, underscoring that clinical reasoning robustness is neither universal across models nor consistent across dental specialties.

The stark contrast between Gemini 2.5 Pro (89.4% GKCQ) and ChatGPT-4o (62.8% GKCQ) underscores the critical importance of domain-specific optimization. This performance gap aligns with Dashti et al.’s [19] findings where fine-tuning ChatGPT-4 with prosthodontics resources boosted accuracy by 10.9% (p < 0.001), confirming that untuned general-purpose architectures fundamentally struggle with dental knowledge structures. As evidenced by Özbay et al. [17] and emphasized by Puleio et al. [20], such models risk significant “hallucinations” (Google Bard: 27.5% misinformation rate vs. tuned ChatGPT-4: 12.5%) and inconsistent performance—a limitation significantly underperformed in ChatGPT-4o’s subpar results. Crucially, Sismanoglu and Capan [16] observed this gap extends to specialty proficiency, with LLMs underperforming by >15% in anatomically complex domains like endodontics versus periodontology. These findings collectively demonstrate that current LLM architectures exhibit systematic deficiencies in dental knowledge encoding, particularly in specialties demanding complex spatial and procedural reasoning. Consequently, specialty-specific validation remains essential before any clinical deployment can be considered.

The marked performance leap of ChatGPT-5 (78.7% overall accuracy), surpassing ChatGPT-4o (61.4%), demonstrates the rapid iterative advancement inherent to large language models (LLMs). This progression aligns with the version-based improvement trend identified by Danesh et al. [21], where ChatGPT-4 outperformed ChatGPT-3.5 by >15% on board-style examination questions. However, ChatGPT-5’s inability to exceed specialized models like Gemini 2.5 Pro—which achieved 93.9% in clinical reasoning tasks—underscores that recency alone is insufficient. This limitation aligns with the evidence [19] that even advanced general-purpose architectures require targeted optimization to bridge the gap in specialized dental knowledge. The performance gap remains consistent across disciplines, with ChatGPT-4o scoring only 46.3% in endodontic decision-making [22]. Models underperformed compared to human benchmarks in anatomically complex domains like endodontics and orthodontics [16]. Collectively, these findings indicate that current LLM architectures exhibit systematic deficiencies, particularly in specialties demanding complex spatial and procedural reasoning.

A hierarchical clustering analysis of stylistic and structural similarities in response patterns provides critical data on technical convergence and divergence within the LLM ecosystem. In our study, the fact that Grok 4 and Gemini 2.5 Pro formed the closest cluster indicates that these models use a similar logical framework in their endodontic reasoning processes. This observation aligns with the finding by Li et al. [22] that the strong performance of Grok 2 and Claude 3.5 in theoretical domains may stem from similar data processing mechanisms. On the other hand, GPT-4o’s position as the most distinct ‘outlier’ in the hierarchical structure and its sharp separation even from its successor, GPT-5, reflects either the uniqueness or the inadequacy of the algorithm it uses to process dental knowledge structures. Puleio et al. [20] emphasized that technological differences between ChatGPT versions have a direct impact on diagnostic accuracy. This behavioral divergence we observed in the hierarchical dendrogram supports the view in the literature [16,22] that systematic shortcomings exhibited by models in dental expertise questions originate from architectural differences. Moreover, this clustering indicates meaningful functional groupings beyond mere performance metrics. The close cluster of Grok 4 and Gemini 2.5 Pro likely reflects shared training data or architectural similarities, making them potentially interchangeable as educational aids and suggesting comparable clinical reasoning pathways. Conversely, GPT-4o’s outlier status highlights divergent reasoning processes—which could represent either innovative processing or less reliable performance in endodontic contexts. From a clinical decision-support perspective, utilizing models from distinct clusters (e.g., combining Gemini 2.5 Pro with Claude 4) may help mitigate shared biases and improve robustness, underscoring that model selection should balance not only accuracy but also architectural diversity to ensure more reliable outcomes.

The findings of this study reveal that large language models (LLMs) tested in the field of dentistry exhibit significant performance differences between clinical decision-making and theoretical knowledge. Models such as Grok 4 (84.3% overall accuracy) and DeepSeek V3 (82.7%) demonstrate statistically higher performance in theoretical knowledge queries (GKCQ) compared to clinical problem-solving (CPQ) scenarios (85.1% vs 81.8% for Grok 4; 84.0% vs 78.8% for DeepSeek V3) indicates that these architectures are strong in knowledge retrieval mechanisms but limited in clinical context integration. In contrast, Claude 4’s markedly higher performance in CPQs (87.9%) compared to GKCQs (81.9%) is consistent with its contextual inference capability in clinical scenarios and validates case-based success findings in the literature [18]. On the other hand, Perplexity’s highest observed performance gap between CPQ-GKCQ (Δ10.1%), consistent with inconsistencies particularly in specific areas such as prosthodontics [23], emphasizes the model’s need for optimization for clinical integration. LLaMA 4’s poor performance in combination-type multiple-choice questions (71.7% overall) reflects deficiencies in its capacity to synthesize visual and textual data [14], which particularly limits its use in clinical settings requiring radiographic interpretation. In conclusion, although the relatively balanced performance of Grok 4 and Claude 4 shows promise for dental education, model-specific limitations must be overcome for the routine use of LLMs as diagnostic decision support tools.

While contemporary LLMs achieved an aggregate accuracy of 78.7% on DUS endodontics questions, performance variability across models and question types highlights the importance of systematic evaluation. As demonstrated by Özbay et al. [17], specialized or fine-tuned models consistently outperform general-purpose architectures (p = 0.004), suggesting that domain-specific optimization is critical for reliable educational and clinical applications [24].

Equally critical is the accountability void in AI-mediated decisions. Current legal frameworks provide no mechanism for assigning liability when LLM-generated treatment recommendations lead to adverse outcomes, as medical responsibility remains exclusively with licensed practitioners [1]. This ethical lacuna is exacerbated by LLMs’ inability to comply with region-specific dental regulations—demonstrated when ChatGPT-4 recommended non-compliant antibiotic protocols in 38% of simulated Türkiye DUS cases [25].

Most fundamentally, contextual deficiency remains an insurmountable gap: text-based success does not equate to clinical competence. As Sismanoglu and Capan [16] empirically demonstrated, LLMs underperformed humans by >15% in domains requiring tactile interpretation (e.g., endodontic file selection) and visual-spatial reasoning (e.g., radiographic caries localization). Real-world practice integrates sensory data, patient affect interpretation, and proprioceptive feedback—dimensions irreducibly beyond textual representation [22]. This limitation is magnified in culturally sensitive contexts where non-verbal cues significantly influence treatment decisions [26].

Despite high statistical accuracy (80–90%), the clinical significance of these LLMs must be interpreted cautiously. In high-stakes postgraduate education, such success indicates a strong knowledge base but lacks the ‘zero-error’ reliability required for autonomous clinical support. Furthermore, as demonstrated in holistic DUS evaluations [16,27], AI models—while exceeding passing thresholds—still struggle with the integrative reasoning of top human specialists. Consequently, LLMs should currently serve as supervised supplementary tools for knowledge reinforcement, particularly in complex fields like endodontics where technical errors have irreversible consequences.

However, this research is subject to several limitations. First, regarding scope, the study focuses exclusively on 127 endodontic questions rather than the full multidisciplinary DUS exam; thus, these results should be viewed as a discipline-specific benchmark. Second, regarding data availability, the non-publication of DUS questions post-2022 created a chronological deficit. Moreover, as 2012–2021 questions are publicly accessible, the potential for ‘data contamination’ exists, where high accuracy may reflect memorization rather than genuine zero-shot clinical reasoning. Third, the study design did not account for the inherent stochasticity of LLMs. Each model was queried only once, whereas standardized benchmarking typically involves multiple iterations (e.g., n = 3 or n = 5) to assess consistency and mitigate stochastic variance. Additionally, querying models via web interfaces instead of APIs limited reproducibility due to dynamic system prompts and the lack of ‘Temperature’ control. Fourth, the evaluation was restricted to a zero-shot prompting strategy. While this ensures a standardized baseline, structured prompting—such as assigning expert roles or ‘Chain-of-Thought’ reasoning—can significantly enhance accuracy [19,22]. For instance, providing domain-specific context has been shown to improve ChatGPT-4’s performance in dental exams by 10.9% [19]. Consequently, our zero-shot approach highlights inherent knowledge retrieval but may underrepresent the models’ full potential as optimized clinical decision-support tools [24]. Fifth, by focusing strictly on the selection of the correct multiple-choice option, this methodology treated LLMs as ‘black boxes.’ This does not account for ‘spurious reasoning’ or hallucinations, where a model might reach the correct answer through flawed logic. Future research should involve expert clinicians to perform qualitative evaluations of the generated rationales. Finally, the textual simulation of radiographic imaging precluded the assessment of multimodal processing capacities. Moreover, performance variations (e.g., Gemini vs. LLaMA) may be influenced by ‘language bias,’ as Turkish linguistic proficiency varies across different models’ training corpora. Despite these limitations, this study remains a pioneering investigation into LLM performance within the context of Turkish dental education.

## Conclusion

While contemporary LLMs demonstrate high accuracy in retrieving and resolving historical exam scenarios, this performance likely reflects advanced memorization and data recall capabilities rather than genuine, autonomous clinical reasoning due to potential data contamination. Consequently, the effective integration of these technologies into clinical practice remains strictly dependent on model architecture, specialty-specific optimization, and rigorous human oversight mechanisms. As digital simulations and image analysis support student education, and LLM-assisted tools improve clinician time management, the reliable implementation of these systems necessarily requires human control, data accuracy, and contextual clinical assessment. In future perspective, LLMs and other AI systems should be positioned not as autonomous diagnostic tools, but as auxiliary instruments that provide case simulations in student education, offer rapid literature access to clinicians, and flag potential inconsistencies in treatment plans based on established protocols.
